# Can patient-reported outcomes be used instead of clinician-reported outcomes and photographs as primary endpoints of late normal tissue effects in breast radiotherapy trials? Results from the IMPORT LOW trial

**DOI:** 10.1016/j.radonc.2019.01.036

**Published:** 2019-05

**Authors:** Indrani S. Bhattacharya, Joanne S. Haviland, Penelope Hopwood, Charlotte E. Coles, John R. Yarnold, Judith M. Bliss, Anna M. Kirby, IMPORT Trialists

**Affiliations:** a The Institute of Cancer Research Clinical Trials and Statistics Unit (ICR-CTSU), United Kingdom; b Cambridge University, Department of Oncology, United Kingdom; c The Institute of Cancer Research, Radiotherapy and Imaging, United Kingdom; d Royal Marsden NHS Foundation Trust and Institute of Cancer Research, Radiotherapy and Imaging, United Kingdom

**Keywords:** Breast, Radiotherapy, Trials, Normal tissue effects, PROMs, Patient-reported

## Abstract

**Background:**

In an era of low local relapse rates after adjuvant breast radiotherapy, risks of late normal-tissue effects (NTE) need to be balanced against risk of relapse. NTE are assessed using patient-reported outcome measures (PROMs), clinician-reported outcomes (CRO) and photographs. This analysis investigates whether PROMs can be used as primary NTE endpoints in breast radiotherapy trials.

**Methods:**

Analyses were conducted within IMPORT LOW (ISRCTN12852634) at 2 and 5 years. NTE were recorded by CRO, photographs and PROMs. Measures of agreement tested concordance, risk ratios for radiotherapy groups were compared, and influence of baseline characteristics on concordance investigated.

**Results:**

In 1095 patients who consented to PROMS and photographs, PROMs were available at 2 and/or 5 years for 976 patients, of whom 909 had CRO and 844 had photographs. Few patients had moderate/marked NTE, irrespective of method used (eg. 19% patients and 9% clinicians reported breast shrinkage at year-5). Patients reported more NTE than assessed from CRO or photographs (*p* < 0.001 for most NTE). Concordance between assessments was poor on an individual patient level; eg. for year-5 breast shrinkage, % agreement = 48% and weighted kappa = 0.17. Risk ratios comparing radiotherapy schedules were consistent between PROMs and CRO or photographs.

**Conclusions:**

Few patients had moderate/marked NTE irrespective of method used. Patients reported more NTE than CRO and photographs, therefore NTE may be underestimated if PROMs are not used. Despite poor concordance between methods, effect sizes from PROMs were consistent with CRO and photographs, suggesting PROMs can be used as primary NTE endpoints in breast radiotherapy trials.

## Highlights

• Few patients reported moderate/marked normal tissue effects (NTE) irrespective of assessment method used.
• Patients reported more NTE than clinician-reported outcomes or photographs.
• Concordance was poor on an individual patient level.
• Treatment comparisons were consistent between assessment methods.

In the current era of low local relapse rates after adjuvant breast radiotherapy [1], [2], the risks of radiotherapy-related late normal-tissue effects (NTE) need to be carefully balanced against the benefits of treatment, requiring detailed collection of NTE data in breast radiotherapy trials. Furthermore, with improvements in breast radiotherapy techniques, including the introduction of intensity-modulated [3] and partial-breast radiotherapy [1], the NTE event rate has also fallen substantially. Consequently, measuring NTE is becoming increasingly challenging.

NTE have been variously assessed in breast radiotherapy trials using clinician-reported outcomes (CRO), photographs and patient-reported outcome measures (PROMs) [2], [3]. The optimal NTE data collection method is unclear and there is no gold standard. The methodology of each assessment type differs. For example, patients may be asked to assess changes in their treated breast since their breast cancer treatment, whereas clinicians compare the patient’s treated and contralateral breasts. Also, the scales used for scoring the different assessments vary.

Irrespective of differences between the methods, the priorities for breast radiotherapy trials are that the method used to detect NTE should be able to differentiate between randomised treatment groups (if a difference exists), and that the information obtained is clinically relevant to patients. Data from breast radiotherapy trials demonstrate that PROMs are able to differentiate between dose/volume regimens [1] and between small dose differences in hypofractionated regimens [2]. PROMs also provide the patients’ perceptions of the impact of their cancer and the consequences of treatment [4] within the framework of the question asked. This analysis investigates within the context of the IMPORT LOW partial-breast radiotherapy trial, (i) the degree of concordance on an individual patient level between PROMs and CRO or photographs, (ii) whether results for the randomised comparisons obtained from PROMs are consistent with those using CRO or photographs and (iii) the influence of baseline characteristics on concordance, with the overall aim of assessing whether PROMs could be used as primary NTE endpoints in future breast radiotherapy trials.

## Methods

### Patient population

IMPORT LOW (ISRCTN12852634) is a multicentre randomised phase III non-inferiority trial comparing safety and efficacy of standard whole-breast radiotherapy with two experimental schedules (reduced-dose and partial-breast radiotherapy) in women with low-risk breast cancer after breast conserving surgery [1].

IMPORT LOW included a comprehensive and systematic investigation of NTE including CRO in all participants, and PROMs and photographs in a subset of patients for which full details of patients and procedures have been published [1]. All centres were invited to participate in the PROMs and photographic sub-studies (until sufficient accrual was achieved). All patients at these centres were invited to participate in the sub-studies until the designated sample size for each sub-study was obtained.

### Procedures

Patients who consented to the PROMs sub-study completed the EORTC QLQ-C30 core questionnaire and QLQ-BR23 breast-specific module [5], [6] and 10-item Body Image Scale (BIS) [7], all of which asked patients to consider their symptoms during the past week. The Hospital Anxiety and Depression Scale [HADS] [8] and protocol-specific questionnaire items relating to ‘change in breast appearance’, ‘breast hardness/firmness’, ‘reduction in size of breast’, ‘change in skin appearance’, ‘is the position of the nipple of your affected breast different from the other side’, ‘problem getting a bra to fit’ and ‘shoulder stiffness’ which may have resulted from any prior breast cancer treatments [32] were also completed. All items (with the exception of HADS) were scored on a four-point scale: none, a little, quite a bit, very much (interpreted as none, mild, moderate, marked). Questionnaires were completed at baseline (pre-radiotherapy) and 6 months, 1, 2 and 5 years after radiotherapy. Patients completed PROMs questionnaires alone with no help from clinicians.

For patients participating in the photographic sub-study, photographs were taken at baseline (post surgery but pre-radiotherapy), year-2 and year-5. Change in photographic breast appearance of the ipsilateral breast was assessed at 2 and 5 years compared with the baseline photograph. Breast size and surgical deficit were scored from the baseline photographs on a 3-point scale (small, medium, large). At 2 and 5 years after radiotherapy, breast appearance change (none/mild/marked) was scored on a pair of photographs (one with the patients’ hands on the hips and one with hands raised) in comparison with the baseline photograph. A panel of observers blinded to patient identity, treatment allocation, and radiotherapy centre scored the photographs, the methodology having been validated in the START pilot trial [9].

CRO including breast shrinkage, breast induration, telangiectasia and breast oedema were scored using the contralateral breast as a comparator with a four-point graded scale (none, a little, quite a bit, very much; interpreted as none, mild, moderate, marked) at 1, 2 and 5 years following radiotherapy in all patients. The CRO items were established and validated in the START trials [10]. Clinicians were not blinded to treatment group.

### Statistical analysis

PROMs were paired with the relevant CRO or photograph at 2 and 5 years for the analyses (Table 1).

**Table 1:** Patient reported outcome measures of specific late NTE in the breast and the corresponding clinician and photographic assessment.

Patient reported outcome measure *(resulting from prior breast cancer treatment)*	Clinician assessment *(treated breast compared with contralateral breast)*	Photographic assessment *(change in appearance compared with baseline photograph)*
Has your affected breast become smaller?	Breast shrinkage	–
Has your affected breast become harder/firmer to the touch?	Breast induration*	–
Was the area of your affected breast swollen?	Breast oedema	–
Have you had a problem getting a bra to fit?	Breast shrinkage	–
Has the overall appearance of your affected breast changed compared with the other side?	–	Overall change in breast appearance
Is the position of the nipple of your affected breast different from the other side?	–	Overall change in breast appearance

* Maximum score in and outside the tumour bed was recorded.

The “quite a bit” and “very much” categories were combined for PROMs and CRO as few NTE were scored as “very much”. This resulted in a 3-point scale corresponding to none, a little (mild), quite a bit/very much (moderate/marked). This also enabled direct comparison with photographs, also scored on a 3-point scale.

Agreement between the data ascertainment methods on an individual patient level was assessed using percentage agreement (with 95% confidence interval), weighted kappa statistic (with 95% confidence interval) and Bowker’s test of symmetry [11]. Guidelines for interpreting the value of weighted kappa in terms of the strength of agreement are <0.20: poor; 0.21–0.40: fair; 0.41–0.6: moderate; 0.61–0.8: good; 0.81–1.00: very good [12]. A significance level of ≤0.005 was used to account for multiple testing in all analyses.

Risk ratios comparing each test radiotherapy schedule with the control group were calculated for each NTE endpoint at year-5 and presented in forest plots for the different assessment methods. Results for breast oedema were not included in this comparison as so few events were reported using PROMs and CRO at year-5.

The influence of baseline patient characteristics on concordance was investigated using stratified analyses, and formally assessed in logistic regression models defining a binary outcome as 1 = concordant (same scores for PROMs and CRO/photographs) versus 0 = discordant (different scores). Baseline factors found to be statistically significantly associated with concordance on univariate analysis were tested together on multivariate analysis. Baseline characteristics tested included age, treatment group, breast size and surgical deficit (assessed from baseline photographs), HADS anxiety and depression subscale scores and body image scores.

All analyses were carried out using STATA version 14 based on a database snapshot taken on June 15th 2016 (as per the primary endpoint analysis).

## Results

**Fig. 1:**
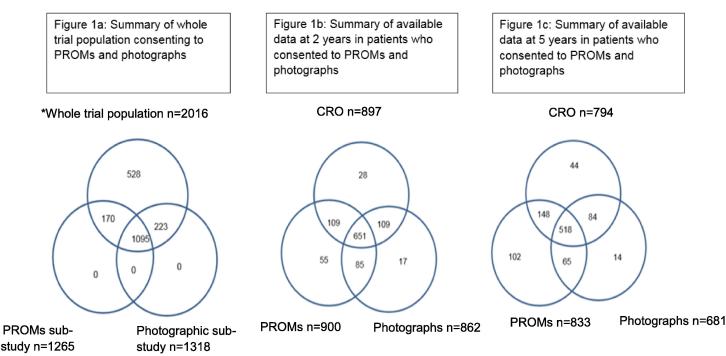
Summary of whole trial population consenting to PROMs and photographs, and data available at 2 and 5 years (^*^Two patients withdrew consent for any of their data to be used in the analysis).

2018 patients were recruited to IMPORT LOW from 71 centres. 2 patients requested exclusion from analyses. In the 41 centres participating in the PROMs sub-study, 1265/1333 (95%) patients consented to PROMs, and 1318/1466 (90%) patients consented to the photographic sub-study from 37 participating centres. 1095 patients consented to both sub-studies (Fig. 1a).

In 1095 patients who consented to both, PROMs were available at 2 and/or 5 years for 976 patients of whom 909 had CRO and 844 had photographs. PROMs, CRO and photographs were available for 651 and 518 patients at year-2 (Fig. 1b) and year-5 respectively (Fig. 1c). Separate analyses were conducted in patients with PROMs and CRO, and PROMs and photographs, at year-2 and year-5. Data regarding baseline characteristics [1] and PROMs questionnaire return rates [13] have been published.

### Overall prevalence of NTE

The overall prevalence of patients with NTE was low, with most scored as none or mild by all three data ascertainment methods (Table 2). Few patients had NTE scored as moderate or marked. NTE which were commonly reported included breast shrinkage, induration and breast appearance change. At year-5, 19% patients and 9% clinicians reported moderate/marked breast shrinkage. With respect to breast induration, 7% patients and 5% clinicians reported moderate/marked changes. For breast appearance change, 18% patients reported moderate/marked changes and photographic assessment reported marked changes in 4%.

**Table 2:** Concordance between PROMs and clinician and photographic assessments of specific NTE at 2 and 5 years in the IMPORT LOW trial.

Patient Reported Outcome	Clinician reported outcome/photograph			% agreement (95% confidence interval)	Weighted Kappa (95% confidence interval)	Bowker’s test of symmetry, *p* value
	None	A little	Quite a bit/very much			
*Breast smaller/shrinkage – 2 years*						
None	276	63	6	400/860;	0.16 (0.16–0.20)	<0.001
A Little	250	105	23	46.5%		
Quite a bit/very much	62	56	19	(43.1–49.9%)		

*Breast smaller/shrinkage – 5 years*						
None	221	60	11	358/751	0.17 (0.14–0.19)	<0.001
A Little	170	115	31	47.7%		
Quite a bit/very much	75	46	22	(44.0–51.3%)		

*Breast harder/induration – 2 years*						
None	432	87	15	493/860	0.11 (0.09–0.12)	<0.001
A Little	202	51	15	57.3%		
Quite a bit/very much	34	14	10	(53.9–60.7%)		

*Breast harder/induration – 5 years*						
None	398	93	21	457/751;	0.12 (0.09–0.19)	0.025
A Little	126	53	10	60.9%		
Quite a bit/very much	32	12	6	(57.3–64.4%)		

*Breast swollen/oedema – 2 yrs*						
None	741	44	5	750/854	0.15 (0.10–0.18)	0.990
A Little	43	9	3	87.8%		
Quite a bit/very much	6	3	0	(85.4–89.9%)		

*Breast swollen/oedema – 5 yrs*						
None	670	24	1	673/743;	0.05 (0.01–0.11)	0.06
A Little	39	3	1	90.6%		
Quite a bit/very much	5	0	0	(88.2–92.6%)		

*PRO-Bra fitting/CRO shrinkage – 2 yrs*						
None	464	161	22	504/860;	0.11 (0.06–0.13)	<0.001
A Little	98	33	18	58.6%		
Quite a bit/very much	26	31	7	(55.2–61.9)		

*PRO-Bra fitting/CRO shrinkage – 5 yrs*						
None	356	145	29	421/752	0.15 (0.08–0.16)	<0.001
A Little	81	52	22	56.0%		
Quite a bit/very much	30	24	13	(52.4–59.6)		

Overall change in appearance*– 2 years						
None	158	9	3	193/731;	0.03 (0.01–0.03)	<0.001
A Little	406	29	4	26.4%		
Quite a bit/very much	97	19	6	(23.2–29.8%)		

Overall change in appearance*– 5 years						
None	138	15	2	199/571;	0.09 (0.05–0.14)	<0.001
A Little	262	48	6	34.9%		
Quite a bit/very much	60	27	13	(30.9–38.9%)		

Nipple position/change in appearance*– 2 years						
None	412	30	4	430/728;	0.04 (0.03–0.05)	<0.001
A Little	191	17	8	59.1%		
Quite a bit/very much	56	9	1	(55.4–62.7%)		

Nipple position/change in appearance*– 5 years						
None	279	48	10	314/569;	0.08 (0.03–0.11)	<0.001
A Little	142	28	4	55.2%		
Quite a bit/very much	37	14	7	(51.0–59.3%)		

* Change in appearance assessed on photograph

### Reporting of NTE by patients compared with either CRO or photographs

**Fig. 2:**
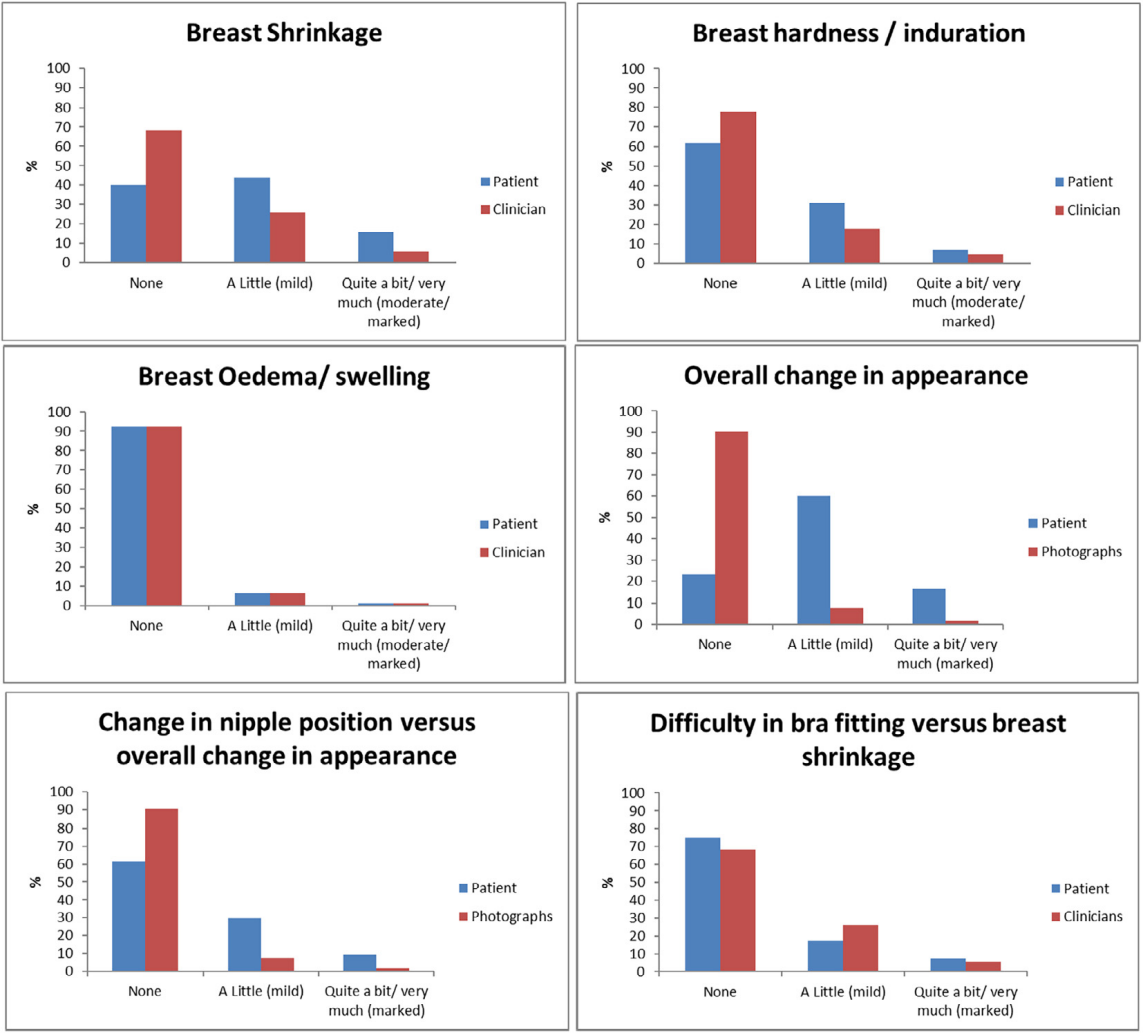
Comparison of year-2 patient reported outcome measures, clinician and photographic assessments of specific late NTE in IMPORT LOW.

**Fig. 3:**
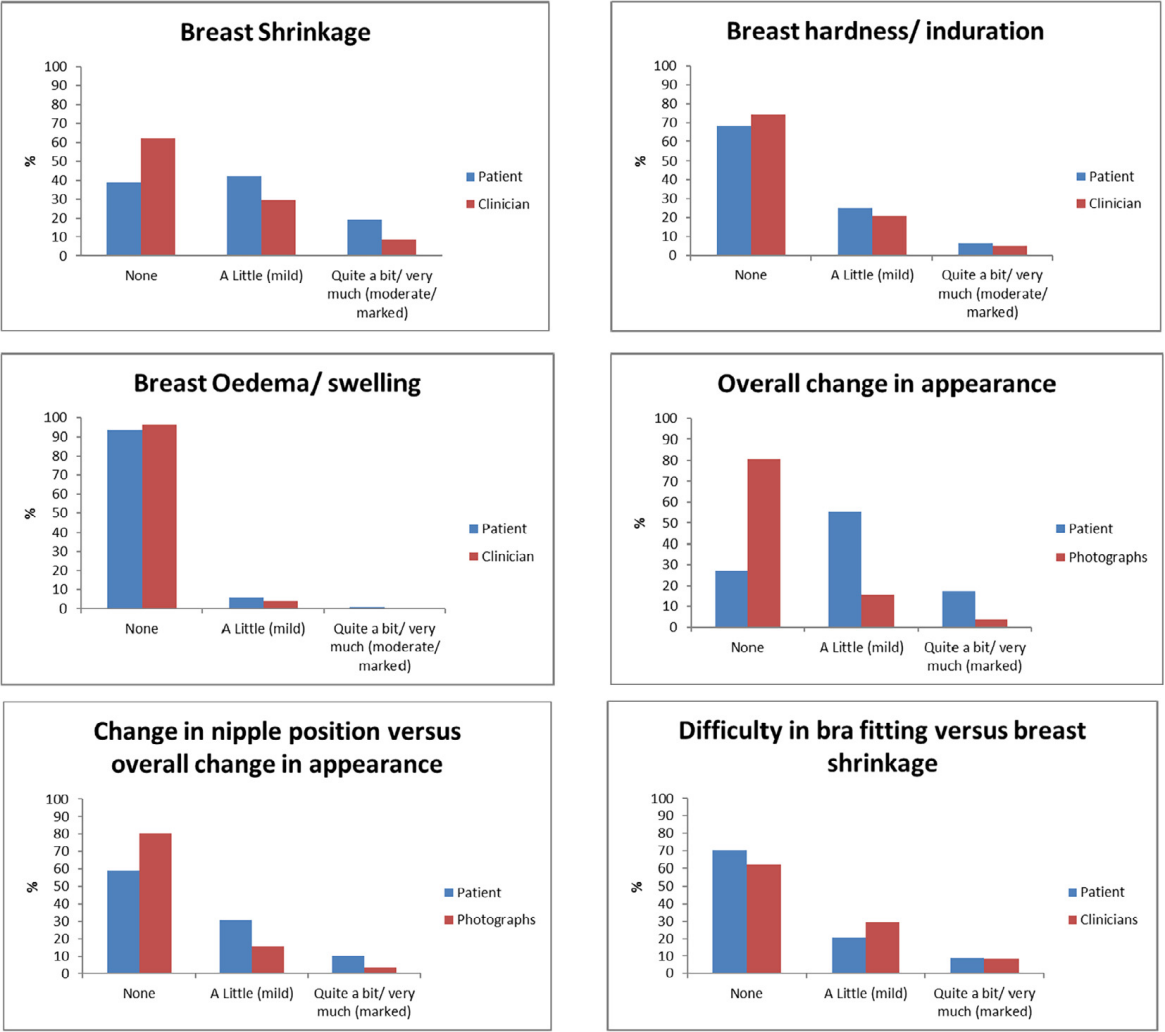
Comparison of year-5 patient reported outcome measures, clinician and photographic assessments of specific late NTE in IMPORT LOW.

Patients reported a higher prevalence of breast changes than CRO and photographs for all NTE assessed, except for more clinically reported mild breast shrinkage compared with patient-reported bra fitting at both time-points (Fig. 2, Fig. 3). Patients and clinicians reported similar prevalences of breast oedema, with very few events at 2 and 5 years. Concordance between PROMs and CRO or photographs of corresponding NTE on an individual patient basis was generally poor (Table 2).

For breast shrinkage at year-5, patients reported more effects than clinicians (Fig. 3); percentage agreement was 48% and concordance was poor as evidenced by the low weighted kappa (0.17, Table 2). Bowker’s test of symmetry was also highly significant (*p* < 0.001) indicating discordance, with patients reporting more effects than clinicians (Table 2). With regard to 5-year breast appearance change, patients reported more NTE than scored on photographs (Bowker’s test of symmetry <0.001) [Table 2]. Agreement was poor (35%), as was concordance (weighted kappa 0.09) [Table 2].

In contrast, for breast induration at year-5, PROMs and CRO appeared better aligned with similar levels of effects reported by both (Fig. 3) and a higher % agreement (61%, Table 2), but concordance remained poor (weighted kappa 0.12, Table 2). In addition, Bowker’s test for symmetry was no longer significant (*p* = 0.025), implying similar effects reported by PROMs and CRO (Table 2).

### Comparison of radiotherapy schedules using PROMs, CRO and photographs

**Fig. 4:**
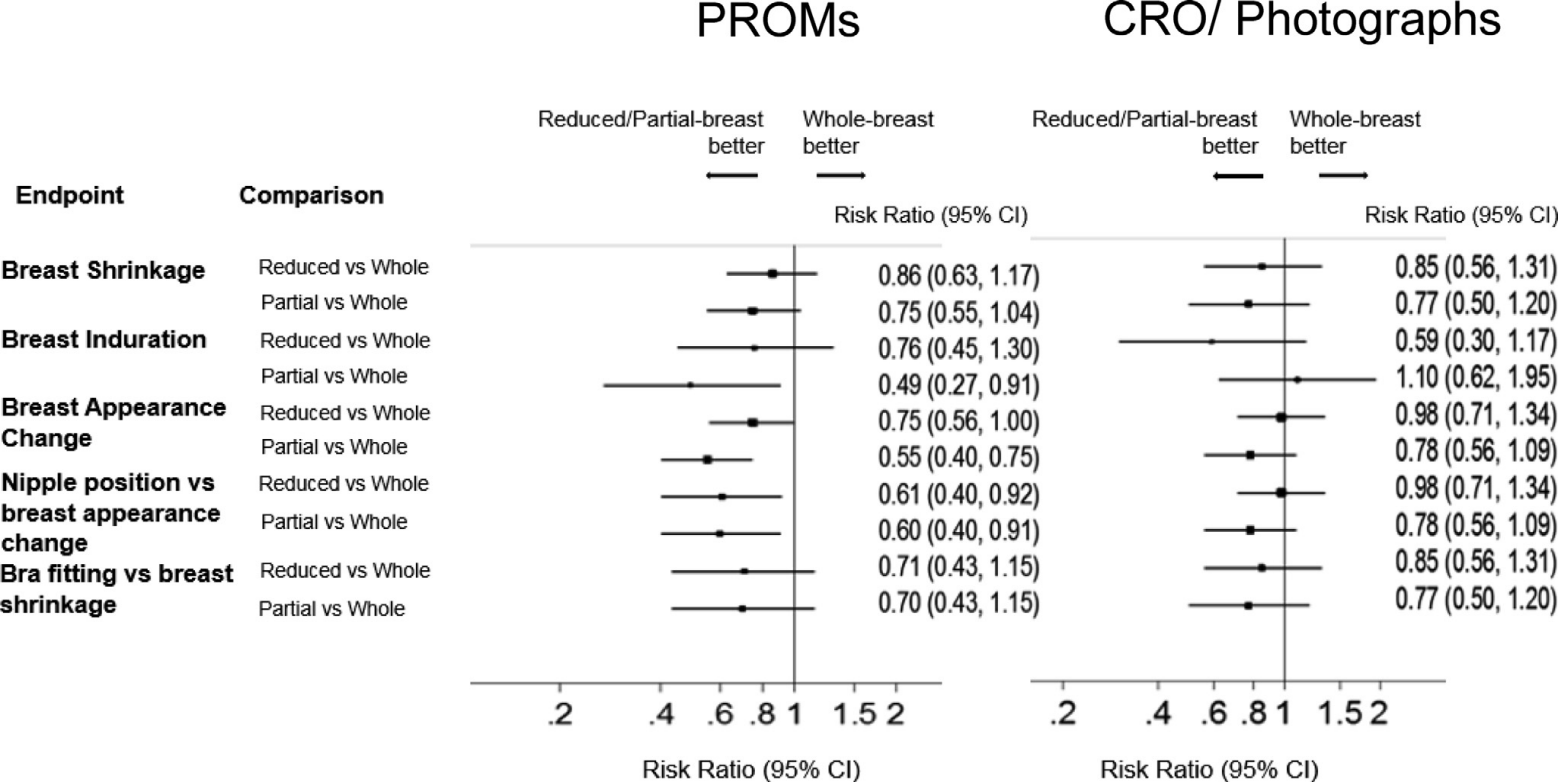
Comparison of the estimates of effect sizes for the randomised radiotherapy groups between PROMs and CRO/photographs at 5 years.

On comparison of the risk ratios for the radiotherapy schedules, similar effect sizes were seen for breast shrinkage and breast appearance change when the analogous question was asked of the patient, or ascertained from either CRO or photographs (Fig. 4). There was some evidence of differing effect sizes between the assessment methods for breast induration, but the confidence intervals overlapped (Fig. 4).

### Associations between baseline characteristics and concordance

On stratified analyses, there was little evidence that concordance varied according to baseline characteristics at 2 or 5 years (Appendix Table A1 & Table A2). Some baseline factors were significantly associated with concordance of PROMs and either CRO or photographs for certain NTE in logistic regression models, but predominantly on univariate analysis only and not across both time-points (Appendix Table A3). For example, larger surgical deficit was associated with discordance of breast shrinkage at year-5 only [OR 0.32 (95%CI 0.16–0.65)] (Appendix Table A3).

## Discussion

This analysis in the context of a randomised trial of partial-breast radiotherapy found few patients had moderate/marked NTE, irrespective of the data ascertainment method used. In general, patients reported more NTE compared with clinicians and photographs. Concordance was poor between PROMs and either CRO or photographs on an individual patient level. However, results obtained for randomised comparisons between treatment groups were consistent for PROMs and either CRO or photographs. There were no clinically significant associations found between baseline characteristics and concordance of NTE.

The low overall prevalence of moderate/marked NTE, irrespective of the data ascertainment method used, has been reported in a number of adjuvant breast radiotherapy trials [1], [2], [13]. It is therefore increasingly important, in an era of improving radiotherapy techniques to monitor NTE using sufficiently sensitive methods. Within IMPORT LOW, patients reported more NTE compared with clinicians or photographs; this has been previously documented in the literature [14], [15], [16], [17], [18], [19], [20], [21]. This suggests NTE may be underestimated if only clinician-reported or photographic outcomes are used. In contrast, the Cambridge IMRT trial [22] found clinicians reported a higher prevalence of breast changes than patients which may be related to the Cambridge study being a single-centre study with assessments conducted by one individual.

Concordance was poor on an individual patient level in IMPORT LOW. This could be explained by, firstly, the methods not being designed to be interchangeable given the different comparators used. Secondly, each method is also asking a slightly different question; when patient-reported bra fitting was compared with clinician-reported breast shrinkage, patients were deciding what a reasonable fit is in general, whereas clinicians reported degree of breast shrinkage. Thirdly, each method has its own scoring sub-scale which may be worded and categorised differently. Poor concordance has been consistently reported in the literature to date [14], [15], [16], [22], [23], [24], [25]. Furthermore, it has been argued that some variation is ‘quite acceptable and comprehensible’ due to the methodological differences between toxicity scoring by patients and clinicians [26].

Although concordance was poor on an individual patient level, the three methods generated similar estimates of effect sizes in terms of comparisons between the randomised treatments, suggesting it is reasonable to use any method. These findings are consistent with those from the START trials [14]. Within IMPORT LOW there also appeared to be a higher sensitivity of PROMs to treatment volume, although the effect sizes obtained from PROMs remained consistent with CRO and photographs. It should be noted that the PROMs investigated in this analysis and the START trials were the protocol-specific items, which were specifically developed to capture late radiotherapy effects [32], rather than generic PROMs related to general quality of life [5].

With respect to the influence of baseline characteristics on concordance, findings were not consistent across NTE or years of assessment and most associations found were significant on univariate analysis only. It is therefore not possible to draw any firm conclusions from these data. The START [14] and Cambridge IMRT [22] trials found no evidence of associations between baseline factors and concordance of NTE assessment methods.

In relation to which NTE assessment methods to use in future breast radiotherapy trials, each has advantages and disadvantages. Clinicians are able to assess the breast with a 3-D view whereas this is not possible with standard photographs (unless taken from various angles providing an overall composite of the breast, although limited resources may prevent this). However, there is a risk of ‘bias reporting’, as clinicians cannot be blinded to the allocated radiotherapy treatment. Also, varying thresholds of experience in grading toxicity between clinicians can lead to interobserver variability; there was no formal training protocol for clinicians assessing NTE in IMPORT LOW. Furthermore, changes in UK working practices including earlier discharge of patients back to primary care make hospital-based follow-up challenging [27].

Obtaining photographs is also becoming increasingly challenging. Firstly, despite consenting to participate in a photographic sub-study, patients may not attend for photographs. There is a risk of ‘informative censoring’ where patients may choose not to attend for photographs either (1) because they do not think there is a problem with their treated breast or (2) they may have experienced NTE and feel uncomfortable about having photographs, resulting in a self-selected population. Of note there was no evidence of change in attendance for year-5 photographs based on year-2 photograph scores in IMPORT LOW. Additionally, workforce changes including closure of medical photography departments make it harder to schedule photographs. It should be noted that photographs provide the only unbiased comparison of NTE between randomised treatment groups [22], [1], [2], [3] as the panel of clinicians scoring photographs are blinded to treatment allocation. Photographs also provide a permanent record at a fixed time point and can be filed and stored for future use. Scoring can also be validated by repeat scoring from different observers [9]. However, in IMPORT LOW, there was a large discrepancy in rating overall change in breast appearance between photographs and PROMs (% agreement = 26% and 35% at year-2 and 5 respectively). Patients reported significantly more NTE at both time-points, suggesting photographs may not capture the changes which are important for patients.

PROMs provide an opportunity to understand the patients’ own perception of NTE within the framework of questions asked. We know that patients report more NTE than clinicians [14], [15], [16], [17], [18], [19], [20], [21] or photographs and therefore, without the use of PROMs, the prevalence of NTE may be underestimated. Furthermore, PROMs are able to distinguish between treatment groups [1], [2]. Within the START trials, all three data ascertainment methods were able to differentiate between randomised treatment groups [2], [28], [29] whereas in IMPORT LOW it was found that only PROMs were able to distinguish between randomised comparisons [1]. This difference in findings is likely related to the NTE event rate being lower in IMPORT LOW than in the START trials. In future breast radiotherapy trials (with expected low NTE rates), PROMs may have better capability in differentiating between treatment groups.

However, there are a number of issues related to PROMs. Firstly, certain patient groups may not wish to participate in a PROMs study resulting in a trial population unrepresentative of the general population. Secondly, obtaining complete datasets can be challenging [4] as questionnaires may not be returned and individual questions may not be completed. Thirdly, there is a risk of bias related to questionnaire return as patients who return questionnaires may have different characteristics to those who don’t and may report either more or fewer side-effects. In IMPORT LOW, women who declined participation in the PROMs sub-study were slightly older than those who did consent [13]. There were no significant differences in the majority of baseline characteristics in those who did or did not return questionnaires at 5 years, with the exception of higher baseline HADS anxiety and depression subscale scores in patients who did not return their year-5 questionnaire [13]. Also, patients who reported more adverse effects at year-2 were more likely to return questionnaires at year-5 [13]. The prevalence of NTE at individual time-points may therefore be overestimated. Finally, irrespective of missing data, there is also risk of ‘bias reporting’, as patients cannot be blinded to treatment group in radiotherapy trials. Although the risk of bias reporting cannot be avoided, strategies can be implemented to reduce missing data, including collecting data electronically, such as via smart phone/email. Reducing numbers of questions in PROM questionnaires to include only the most salient and discriminating questions may also improve return rates. As well as obtaining complete and unbiased data-sets for PROMs, improvements in the standardisation of analysis, interpretation and reporting of PROMs data in clinical trials are also required to enable cross-comparison of data between trials [30].

We have discussed whether PROMs could potentially replace either CRO or photographs to assess NTE. Broadly, patients rate their subjective satisfaction with an experience of a range of breast changes, whilst clinicians seek objective adverse treatment effects. Therefore, the differences and agreements found by the methods contribute to the overall trial evaluation from multiple perspectives, affecting both the individual patient and randomised trial population. We acknowledge CRO are still widely supported and an alternative viewpoint is that both PROMs and CROs may be necessary as they measure differing aspects of disease experience and are complementary [31].

The main limitation of this analysis is that the IMPORT LOW trial was not designed to address the specific question of concordance between the data ascertainment methods therefore methodological issues regarding data ascertainment exist. These include each of the methods asking a slightly different question and using different comparators, with various subscales. The lack of standardisation between the methods may limit comparability between PROMs and either CRO or photographs.

Few patients had moderate/marked NTE irrespective of method used. Patients reported more NTE than CRO and photographs, therefore NTE may be underestimated if PROMs are not used. Despite poor concordance between assessment methods, effect sizes from PROMs were consistent with CRO and photographs, suggesting PROMs can be used as primary NTE endpoints in breast radiotherapy trials.

## Conflicts of interest

JMB discloses Research Funding: AstraZeneca, Merck Sharp & Dohme, Medivation, Puma Biotechnology, Clovis Oncology, Pfizer, Janssen-Cilag, Novartis, Roche. All other authors have no conflicts of interest.

## Figures and Tables

**Table A1:** Concordance between PROMs and clinician and photographic assessments of specific NTE at year-2 stratified by baseline characteristics in the IMPORT LOW trial.

Baseline item	Breast shrinkage		Breast induration		Breast Swelling		Overall change in appearance		Nipple position		Bra fitting	
	%agreement (95% CI)	Weighted kappa (95% CI)	% agreement (95% CI)	Weighted kappa (95% CI)	% agreement (95% CI)	Weighted kappa (95% CI)	% agreement (95% CI)	Weighted kappa (95% CI)	% agreement (95% CI)	Weighted kappa (95% CI)	%agreement (95% CI)	Weighted kappa (95% CI)
*Age*												
<60 years	43.7 (37.9–49.6)	0.12 (0.03–0.17)	51.2 (45.3–57.1)	0.06 (0.02–0.06)	87.6 (83.2–91.2)	0.07 (−0.001–0.11)	24.2 (18.9–30.1)	0.03 (0.01–0.04)	61.5 (55.0–67.7)	0.06 (0.02–0.10)	57.5 (51.6–63.3)	0.08 (0.001–0.14)
≥60 years	48.0 (43.8–52.2)	0.18 (0.14–0.21)	60.5 (56.3–64.5)	0.14 (0.09–0.18)	87.9 (85.0–90.5)	0.19 (0.17–0.29)	27.5 (23.6–31.7)	0.02 (0.02–0.03)	57.9 (53.4–62.3)	0.04 (–0.001–0.08)	59.2 (55.0–63.2)	0.13 (0.10–0.16)

*Treatment Group*												
Group 1 (whole-breast)	49.1 (43.1–55.1)	0.23 (0.18–0.35)	48.6 (42.6–54.6)	0.03 (0.01–0.12)	86.7 (82.2–90.5)	0.11 (–0.03–0.19)	25.7 (20.3–31.8)	0.03 (0.02–0.06)	57.9 (51.3–64.3)	0.04 (0.03–0.07)	53.8 (47.7–59.8)	0.09 (0.02–0.19)
Group 2 (reduced-dose)	45.2 (39.5–51.1)	0.12 (0.07–0.14)	60.1 (54.3–65.8)	0.16 (0.08–0.26)	86.0 (81.5–89.8)	0.12 (0.04–0.20)	26.7 (21.3–32.7)	0.03 (0.002–0.04)	57.1 (50.7–63.3)	0.05 (–0.009–0.11)	63.2 (57.4–68.7)	0.11 (0.06–0.21)
Group 3 (partial-breast)	45.3 (39.4–51.3)	0.14 (0.13–0.19)	63.0 (57.1–68.7)	0.14 (0.09–0.20)	90.8 (86.8–93.9)	0.25 (0.15–0.25)	26.7 (21.3–32.7)	0.02 (0.01–0.04)	62.2 (55.8–68.3)	0.03 (0.004–0.12)	58.5 (52.6–64.3)	0.12 (0.09–0.17)

*Breast size*												
Small	47.6 (41.9–53.3)	0.17 (0.14–0.18)	58.3 (52.5–63.8)	0.05 (0.03–0.12)	90.5 (86.6–93.5)	−0.05 (–0.06–0.04)	24.2 (19.6–29.2)	−0.003 (−0.02– –0)	55.6 (50.0–61.0)	0.01 (−0.004–0.03)	56.8 (51.1–62.4)	0.07 (0.05–0.13)
Medium	48.8 (42.2–55.2)	0.21 (0.19–0.27)	58.8 (52.3–65.0)	0.09 (0.06–0.15)	86.6 (81.6–90.7)	0.16 (0.07–0.24)	24.3 (19.2–30.1)	0.02 (–0.01–0.04)	63.5 (57.3–69.4)	0.10 (0.04–0.14)	60.5 (54.0–66.7)	0.15 (0.05–0.25)
Large	53.7 (44.9–62.3)	0.27 (0.27–0.37)	54.0 (45.3–62.6)	0.11 (0.05–0.16)	80.4 (72.8–86.7)	0.20 (0.12–0.31)	33.1 (25.4–41.5)	0.10 (0.06–0.14)	59.9 (51.3–68.0)	0.08 (0.08–0.15)	55.1 (46.4–63.7)	0.15 (0.13–0.27)

*Surgical deficit*												
Small	53.4 (48.7–58.1)	0.20 (0.16–0.23)	61.1 (56.4–65.6)	0.10 (0.07–0.21)	86.9 (83.5–89.9)	0.18 (0.14–0.30)	29.8 (25.7–34.1)	0.004 (–0.02–0.03)	66.9 (62.5–71.1)	0.02 (0.01–0.04)	66.0 (61.5–70.3)	0.15 (0.13–0.21)
Medium	41.9 (34.6–49.5)	0.12 (0.08–0.27)	53.0 (45.5–60.5)	0.04 (–0.05–0.08)	86.0 (80.0–90.7)	0.003 (–0.08–0.05)	19.1 (13.8–25.5)	0.03 (0.01–0.06)	44.4 (37.1–51.8)	0.02 (0.001–0.08)	38.9 (31.7–46.4)	–0.06 (0.15– –0.04)
Large	37.0 (24.3–51.3)	0.17 (0.06–0.24)	44.6 (31.3–58.5)	0.02 (–0.05–0.25)	92.6 (82.1–97.9)	0.27 (0–0.43)	17.5 (8.7–29.9)	0.03 (0.003–0.09)	43.6 (30.3–57.7)	0.05 (0.04–0.08)	50.0 (35.8–64.2)	0.28 (0.25–0.31)

*HADs anxiety*												
0–7 (normal)	46.6 (42.8–50.4)	0.15 (0.11–0.16)	59.8 (56.0–63.5)	0.12 (0.06–0.19)	88.8 (86.2–91.1)	0.16 (0.08–0.24)	26.8 (23.2–30.6)	0.02 (0.02–0.03)	59.2 (55.1–63.2)	0.03 (0.01–0.05)	62.4 (58.5–66.2)	0.18 (0.14–0.22)
8–10 (borderline)	45.7 (36.4–55.2)	0.16 (0.05–0.26)	49.6 (40.2–59.0)	0.04 (–0.03–0.10)	87.0 (79.4–92.5)	0.15 (0.11–0.28)	23.7 (15.5–33.6)	0.04 (0.01–0.04)	55.9 (45.2–66.2)	0.07 (–0.04–0.11)	55.0 (45.2–64.6)	0.04 (–0.008–0.17)
≥11 (case)	48.2 (34.7–62.0)	0.30 (0.26–0.48)	44.6 (31.3–58.5)	0.06 (–0.09–0.30)	80.0 (67.0–89.6)	**	26.3 (15.5–39.7)	0.006 (–0.04–0.06)	63.2 (49.3–75.6)	0.07 (–0.04–0.11)	66.7 (52.9–78.6)	0.25 (0.14–0.27)

*HADs depression*												
0–7 (normal)	46.5 (43.0–50.1)	0.16 (0.16–0.19)	58.3 (54.8–61.8)	0.12 (0.04–0.14)	88.8 (86.4–90.9)	0.16 (0.07–0.29)	26.9 (23.6–30.4)	0.03 (0.02–0.04)	58.9 (55.0–62.6)	0.04 (0.01–0.06)	59.5 (56.0–62.9)	0.11 (0.09–0.14)
8–10 (borderline)	44.4 (29.6–60.0)	0.16 (0.12–0.28)	44.4 (29.6–60.0)	0.03 (–0.08–0.19)	81.8 (67.3–91.8)	0.20 (0.06–0.28)	23.7 (11.4–40.2)	–0.05 (–0.20—0.001)	65.8 (48.6–80.4)	0.07 (–0.02–0.25)	51.1 (35.8–66.3)	0.08 (0.03–0.19)
≥11 (case)	46.2 (19.2–74.9)	0.19 (–0.29–0.22)	53.8 (25.1–80.8)	0.02 (–0.18–0.08)	61.5 (31.6–86.1)	**	0	–0.07 (–0.50–0.37)	44.4 (13.7–78.8)	0.08 (0–0.24)	30.8 (9.1–61.4)	–0.04 (–0.46–0.02)

*BIS*												
0–10	47.1 (41.8–52.4)	0.10 (0.08–0.13)	65.3 (60.1–70.2)	0.15 (0.07–0.17)	91.1 (87.6–93.8)	0.28 (0.23–0.40)	29.7 (24.6–35.2)	–0.02 (–0.04–0.02)	61.3 (55.6–66.9)	–0.03 (–0.05–0.03)	62.2 (57.0–67.3)	0.04 (–0.03–0.11)
≥11	46.1 (41.7–50.6)	0.20 (0.17–0.26)	51.6 (47.1–56.1)	0.07 (0.02–0.13)	85.5 (82.1–88.5)	0.08 (0.02–0.15)	24.1 (20.2–28.5)	0.05 (0.04–0.06)	57.5 (52.6–62.2)	0.08 (0.04–0.09)	56.0 (51.5–60.4)	0.14 (0.10–0.20)

** Weighted kappa statistic not done as insufficient patient numbers in categories.

**Table A2:** Concordance between PROMs and clinician and photographic assessments of specific NTE at year-5 stratified by baseline characteristics in the IMPORT LOW trial.

Baseline item	Breast Shrinkage		Breast induration		Breast Swelling		Overall change in appearance		Nipple position		Bra fitting	
	% agreement (95% CI)	Weighted kappa (95% CI)	% agreement (95% CI)	Weighted kappa (95% CI)	% agreement (95% CI)	Weighted kappa (95% CI)	% agreement (95% CI)	Weighted kappa (95% CI)	% agreement (95% CI)	Weighted kappa (95% CI)	% agreement (95% CI)	Weighted kappa (95% CI)
*Age*												
<60 years	51.8 (45.5–58.0)	0.27 (0.15–0.32)	55.4 (49.1–61.6)	0.14 (0.04–0.20)	88.3 (83.7–92.0)	0.15 (0.10–0.20)	26.8 (21.1–33.0)	0.03 (0.02–0.04)	54.2 (46.8–61.4)	0.02 (–0.05–0.05)	58.5 (52.8–64.6)	0.10 (0.01–0.13)
≥60 years	45.5 (41.1–50.1)	0.11 (0.07–0.13)	63.7 (59.3–67.9)	0.09 (0.04–0.17)	91.8 (89.0–94.1)	–0.04 (–0.04–0.03)	32.5 (28.2–37.0)	0.03 (0.003–0.03)	55.7 (50.5–60.7)	0.10 (0.04–0.17)	63.2 (58.9–67.4)	0.20 (0.14–0.26)

*Treatment group*												
Group 1 (whole-breast)	43.2 (36.6–49.9)	0.13 (0.11–0.18)	55.0 (48.3–61.6)	0.08 (0.01–0.09)	88.1 (83.1–92.0)	0.07 (–0.05–0.13)	36.1 (29.1–43.6)	0.08 (0.005–0.09)	56.4 (48.8–63.7)	0.12 (0.06–0.17)	57.7 (51.4–63.9)	0.13 (0.09–0.16)
Group 2 (reduced-dose)	49.0 (42.8–55.3)	0.21 (0.15–0.23)	62.3 (56.0–68.2)	0.12 (–0.04–0.22)	89.8 (85.4–93.2)	0.02 (–0.05–0.06)	31.6 (25.0–38.7)	0.09 (0.04–0.16)	51.3 (44.0–58.6)	0.07 (0.06–0.15)	62.3 (56.2–68.0)	0.15 (0.04–0.19)
Group 3 (partial-breast)	50.2 (44.0–56.4)	0.17 (0.08–0.20)	64.5 (58.4–70.3)	0.15 (0.10–0.17)	93.5 (89.8–96.2)	0.07 (–0.04–0.17)	36.8 (30.1–43.9)	0.10 (0.07–0.14)	57.8 (50.6–64.7)	0.05 (–0.04–0.11)	64.7 (58.6–70.4)	0.21 (0.07–0.24)

*Breast size*												
Small	46.6 (40.5–52.8)	0.20 (0.17–0.25)	61.6 (55.5–67.4)	0.16 (0.11–0.24)	92.5 (88.6–95.3)	–0.03 (–0.06– –0.003)	29.7 (24.0–35.9)	0.04 (0.02–0.07)	54.4 (47.8–60.8)	0.11 (0.07–0.14)	61.6 (55.7–67.2)	0.19 (0.15–0.23)
Medium	44.9 (37.9–52.0)	0.14 (0.07–0.14)	63.2 (56.2–69.9)	0.13 (0.09–0.33)	90.6 (85.7–94.2)	0.04 (–0.04–0.06)	37.0 (30.3–44.1)	0.11 (0.10–0.14)	55.5 (48.3–62.5)	0.12 (0.09–0.25)	58.7 (51.9–65.2)	0.13 (0.07–0.21)
Large	51.1 (42.3–60.0)	0.17 (0.14–0.20)	59.1 (50.2–67.6)	0.06 (0.01–0.09)	85.5 (78.3–91.0)	0.09 (–0.01–0.16)	39.5 (30.7–48.9)	0.18 (0.15–0.21)	55.6 (46.1–64.7)	0.02 (–0.19–0.08)	61.6 (52.5–70.2)	0.20 (0.14–0.34)

*Surgical deficit*												
Small	52.1 (47.1–57.0)	0.20 (0.15–0.22)	64.5 (59.6–69.1)	0.17 (0.07–0.20)	90.0 (86.6–92.7)	0.04 (–0.05–0.09)	39.4 (34.3–44.5)	0.10 (0.09–0.14)	61.0 (55.8–66.0)	0.05 (0.02–0.08)	66.7 (62.1–71.2)	0.19 (0.11–0.29)
Medium	39.2 (31.3–47.5)	0.09 (0.03–0.17)	55.4 (47.0–63.6)	0.07 (–0.06–0.15)	91.2 (85.4–95.2)	0.09 (–0.02–0.17)	26.4 (19.4–34.4)	0.08 (0.06–0.11)	46.9 (38.6–55.3)	0.09 (–0.02–0.21)	44.7 (36.9–52.7)	0.03 (–0.02–0.07)
Large	26.1 (14.3–41.1)	–0.07 (–0.21–0.06)	56.5 (41.1–71.1)	0.05 (–0.14–0.28)	90.9 (78.3–97.5)	–0.04 (–0.11–0.04)	18.6 (8.4–33.4)	–0.03 (–0.09–0.04)	31.0 (17.6–47.1)	0.02 (–0.16–0.15)	56.5 (41.1–71.1)	0.27 (0.19–0.35)

*HADs anxiety*												
0–7 (normal)	48.3 (44.2–52.4)	0.18 (0.16–0.24)	62.8 (58.8–66.7)	0.09 (0.04–0.12)	91.8 (89.3–93.9)	0.03 (–0.04–0.06)	34.9 (30.5–39.4)	0.07 (0.04–0.13)	55.8 (51.1–60.5)	0.05 (0.03–0.08)	62.4 (58.5–66.2)	0.18 (0.13–0.20)
8–10 (borderline)	45.1 (35.2–55.3)	0.09 (0.06–0.19)	55.9 (45.7–65.7)	0.14 (0.05–0.33)	93.1 (86.2–97.2)	0.18 (0–0.31)	32.4 (21.8–44.5)	0.13 (0.09–0.16)	46.5 (34.5–58.7)	0.05 (0.003–0.13)	55.0 (45.2–64.6)	0.04 (–0.02–0.13)
≥11 (case)	46.3 (32.6–60.4)	0.24 (0.20–0.38)	48.1 (34.0–62.4)	0.17 (0.04–0.21)	71.7 (57.7–83.2)	–0.005 (–0.08–0.16)	38.1 (23.6–54.4)	0.20 (–0.04–0.37)	62.8 (46.7–77.0)	0.31 (0.14–0.47)	66.7 (52.9–78.6)	0.25 (0.16–0.32)

*HADs depression*												
0–7 (normal)	47.8 (44.1–51.6)	0.17 (0.12–0.21)	61.3 (57.5–64.9)	0.10 (–0.004–0.11)	91.6 (89.3–93.5)	0.05 (–0.05–0.14)	34.5 (30.5–38.7)	0.08 (0.06–0.10)	56.1 (51.8–60.4)	0.08 (0.04–0.10)	62.6 (58.9–66.1)	0.17 (0.16–0.19)
8–10 (borderline)	47.8 (32.5–63.3)	0.22 (0.16–0.46)	53.5 (37.7–68.8)	0.23 (0.17–0.35)	76.7 (61.4–88.2)	0.07 (0–0.11)	38.7 (21.8–57.8)	0.21 (0.10–0.29)	41.9 (24.5–60.9)	0.05 (–0.19–0.36)	50 (34.6–65.4)	0.07 (–0.05–0.22)
≥11 (case)	16.7 (4.2–64.1)	–0.33 (–0.43–0)	83.3 (35.9–99.6)	0.40 (0.20–0.42)	66.7 (22.3–95.7)	0 (0–1.0)	50.0 (6.8–93.2)	0.14 (0–0.25)	50.0 (6.8–93.2)	**	17.7 (15.7–84.3)	0.11 (0.07–0.18)

*BIS*												
0–10	53.0 (47.3–58.6)	0.22 (0.14–0.26)	67.2 (61.7–72.4)	0.15 (0.07–0.18)	93.9 (90.6–96.3)	–0.03 (–0.03–0.02)	38.8 (32.6–45.3)	0.06 (0.06–0.12)	61.6 (55.1–67.8)	0.10 (0.03–0.19)	65.6 (60.2–70.7)	0.19 (0.16–0.21)
≥11	43.8 (39.1–48.6)	0.14 (0.10–0.15)	56.3 (51.5–61.0)	0.09 (0.08–0.13)	88.2 (84.8–91.1)	0.07 (0.04–0.21)	32.0 (27.1–37.3)	0.11 (0.08–0.31)	50.6 (45.1–56.1)	0.06 (0.03–0.12)	58.8 (54.2–63.4)	0.11 (0.04–0.14)

** Weighted kappa statistic not done as insufficient patient numbers in categories.

**Table A3:** Summary of baseline factors associated with concordance between PROMs and CRO/photographs using logistic regression models (univariate analysis) at 2 and 5 years in IMPORT LOW.

NTE assessed by PROM vs CRO/photo	Time point	Factor associated with concordance *Odds Ratio (OR) 95% confidence interval (95%CI), p value*
Breast smaller versus shrinkage	2 years	–
	5 years	Larger surgical deficit: 0.32 (0.16–0.65), *p* = 0.001
Breast hardness/firmness versus	2 years	Treatment group 3: 1.81 (1.29–2.53), *p* = 0.001
induration	5 years	–
Breast swelling versus oedema	2 years	Larger breast size: 0.43 (0.24–0.76), *p* = 0.004
	5 years	Case levels of anxiety: 0.23 (0.12–0.44), *p* < 0.001 and borderline depression: 0.30 (0.14–0.65) *p* = 0.002**
Bra fitting versus breast shrinkage	2 years	Medium surgical deficit: 0.33 (0.23–0.47), *p* < 0.001
	5 years	Younger age 1.00 (1.01–1.06), *p* = 0.002
Change in appearance versus	2 years	–
photographic appearance change*	5 years	–
Nipple position affected versus	2 years	Larger surgical deficit: 0.38 (0.22–0.68), *p* = 0.001
photographic appearance change*	5 years	Larger surgical deficit: 0.29 (0.14–0.57), *p* < 0.001

* Comparison with photographic appearance.

** Anxiety and depression were tested on multivariate analysis and higher levels of anxiety (as measured on HADs) remained significantly associated with discordance for breast oedema [OR 0.31, 95%CI 0.15–0.68, *p* = 0.003].
